# Editorial: From the hydrophobic core to the globular-disorder interface: New challenges and insights into protein design

**DOI:** 10.3389/fmolb.2023.1151676

**Published:** 2023-02-06

**Authors:** Sankar Basu, Devlina Chakravarty, Qingzhen Hou, Vladimir N. Uversky

**Affiliations:** ^1^ Department of Microbiology, Asutosh College, University of Calcutta, Kolkata, India; ^2^ National Library of Medicine, National Center for Biotechnology Information, National Institutes of Health, Bethesda, MD, United States; ^3^ Department of Biostatistics, School of Public Health, Cheeloo College of Medicine, Shandong University, Jinan, Shandong, China; ^4^ Department of Molecular Medicine, USF Health Byrd Alzheimer’s Research Institute, Morsani College of Medicine, University of South Florida, Tampa, FL, United States

**Keywords:** protein design, intrinsically disordered proteins (IDPs), disorder—globular interface, mutational scanning, disorder transitions in proteins, prediction of protein-protein binding sites of IDPs, multi-step electron-transfer activity, high throughput approach

The biophysical foundations of protein folding (Dill and MacCallum, 2012) still remain largely unexplained despite the many years of intensive experimental (Dobson, 2004) and computational research (Callaway, 2020; Jumper et al., 2021). It is rightly believed that the most objective way of probing our understanding of the basic principles of protein folding is reflected in the ability to successfully resolve the ‘inverse protein folding” problem (Yue and Dill, 1992); i.e., the capability to rationally design functional proteins, *de novo*. In recent years, the field has undoubtedly become diverse and complex, with the design of fold-switch proteins, proteins with targeted functional modulations etc.—one of the unmet goals of the latter of which is to aid ‘protein therapy’ (Dimitrov, 2012; Blazeck et al., 2022; Svilenov et al., 2022).

**FIGURE 1:**
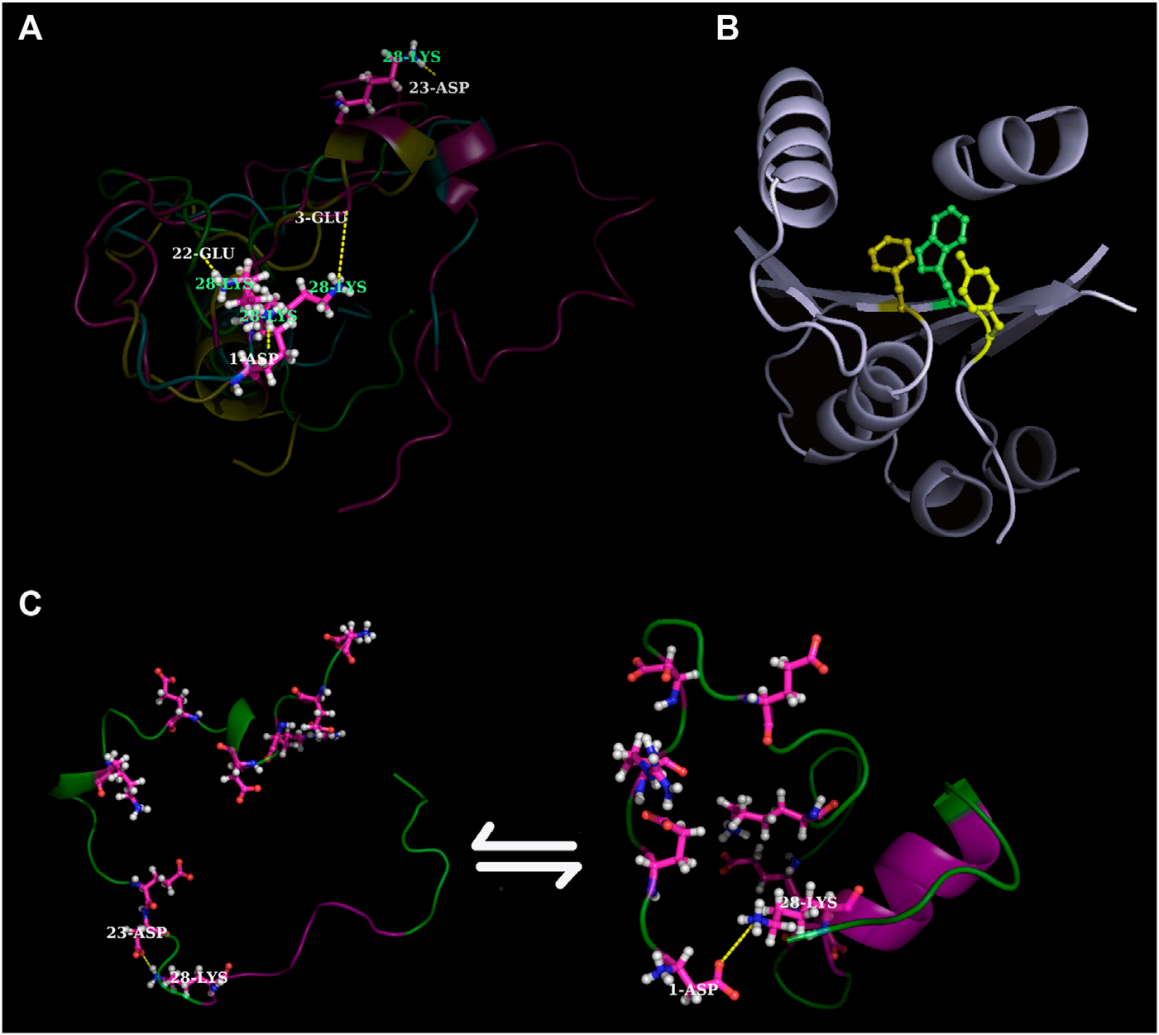
Recent findings in contemporary protein design across the globular—disorder evolutionary interface. **(A)** shows ephemeral salt-bridge dynamics in IDPs (in relation to administering cumulative mutations in probing disorder-to-order transitions in IDPs) (Basu and Biswas, 2018). **(B)** shows a 3-clique (Basu et al., 2011), a ubiquitous packing motif in densely packed cores that heavily influences the formation and sustenance of alternatively packed hydrophobic cores within the same protein fold (Biswas et al., 2022). **(C)** shows how Aβ_42_ temporarily shapes into partial order during its molecular dynamic simulations (Basu and Biswas, 2018). Images in **(A,C)** have been reconstructed from scratch using suitable MD simulation snapshots. In contrast, the image in **(B)** have been reproduced with appropriate copyright permissions from BMC Bioinformatics.

Furthermore, since the discovery of intrinsically disordered proteins (IDPs), with their characteristic binding promiscuity attributed to their physical flexibility, attempts to understand and design protein disorder transitions (Bandyopadhyay and Basu, 2020; Roy et al., 2022) and to address the “globular/ordered–disordered” evolutionary interface (Nagibina et al., 2020) in proteins are among the most intriguing challenges in this field. It is but natural that designs of ordered proteins and IDPs should use different principles and the field is enriching with ideas and findings on both (Figure 1). For example, in globular proteins, a given protein fold (or a hydrophobic core) can be modulated by the alternative modes of side-chain packing (Basu et al., 2011; Biswas et al., 2022), whereas in IDPs, the disorder-to-order transitions can be triggered by cumulative point mutations (Basu and Biswas, 2018). Although there are not yet general evolutionary rules regarding the origin of disordered and globular proteins, instances of successful design (for example) of folded globular repeats from disordered ancestors (Zhu et al., 2016) have raised much hope. A successful endeavor along these directions is of high biophysical and mechanistic importance (Theillet et al., 2013). Furthermore, findings in this area would also serve great therapeutic benefits in the wide array of IDP-related deadly human diseases (Coskuner-Weber and Uversky, 2018).

Themed on protein designs across globular and disordered proteins as well as their evolutionary interface, this Research Topic (RT) currently comprises of five original research articles and a review on the experimental and computational advances in protein design.

In their study of the concentration- and time-dependent effects of a cholesterol-based detergent Chobimalt on insulin amyloid aggregation, a group of Andrey Musatov utilized a multiparametric experimental approach that included a combination ThT fluorescence assay, CD, AFM, SANS, and SAXS (Siposova et al.). This analysis revealed intriguing dose-dependent effect of this amphiphilic compound. The data indicate that not only kinetics but also the morphology of fibrillar aggregates can be dramatically changed in the presence of specific additives.

A group of Bogdan S. Melnik used their experience in the analysis of protein mutants to understand the effect of various amino acid substitutions on the protein energy landscape (Majorina et al.). This knowledge provides means for the rational design of amino acid substitutions that would have the desired effects on the multistate proteins stability and the rates of their native or intermediate state formation. It also can be used for rational design of protein mutants with the desired properties.

A group of Cheng Wang and Dongdong Qiao provided an up-to-date overview of computational methods for prediction of protein-protein binding sites of intrinsically disordered proteins (IDP-PPIS) (Chen et al.). Analysis of 30 recently published IDP-PPIS predictors indicated that based on their underlying principles, they can be divided into three categories, where the algorithms are grounded on the scoring functions, machine learning-based prediction, or consensus approach. Provided information on the details of algorithms and their performances can be used as a guide for selecting different methods.

A group of Ramanathan Sowdhamini utilized extensive computational docking and multi-ns molecular dynamics (MD) simulations to look at the effects of a plant cyclic disulfide-rich peptide (“cyclotide”) isolated from a medicinal plant *Clitoria ternatea* on the conformational dynamics and destabilization of the β-amyloid fibrils, which are well-known hallmarks of Alzheimer’s disease (Kalmankar et al.). This study revealed that the anti-aggregation potential of this cyclic disulfide-rich peptide can be attributed to the cyclotide binding-induced unfolding and opening up of the amyloid β-sheet structure of Aβ fibrils and opened a way for the design of novel cyclotide-based drugs against protein aggregation.

Ennist et al. described a designed electron-transport chain with the multi-step electron-transfer activity based on the *de novo* designed reaction center maquette protein (the RC maquette) that assembles metal ions, tyrosine, a Zn tetrapyrrole, and heme. Although in its apo-form, the RC maquette is characterized by a dynamic and flexible structure, it can predictably fold into a more ordered holo-state upon binding of various cofactors, such as electron donors, pigments, and electron acceptors. The authors believe that the novel and better RC maquettes can be designed with the thermodynamic efficiency that could be substantially higher than that of the natural photosystems.

A group of Raghavan Varadarajan designed a novel high throughput approach for the accurate mapping of interfacial residues based on screening of a query protein against a panel of chemically masked single cysteine mutants of its interacting partner, which are displayed on the surface of yeast cells (Ahmed et al.). In this approach, the binding residues are decrypted by the flow cytometry-based probing of the loss of binding to the labeled cognate partner. This tool was applied for the identification of the interfacial residues for MazEF3, MazEF6 and MazEF9 toxin-antitoxin (TA) systems from *Mycobacterium tuberculosis*, where the toxin has a conserved fold, while the structurally diverse antitoxins have intrinsically disordered toxin-binding regions.

Based on the response from the scientific community across the globe, the Research Topic has been extended for a second year (Volume II).
